# Social-Stress-Responsive Microbiota Induces Stimulation of Self-Reactive Effector T Helper Cells

**DOI:** 10.1128/mSystems.00292-18

**Published:** 2019-05-14

**Authors:** Michal Werbner, Yiftah Barsheshet, Nir Werbner, Mor Zigdon, Itamar Averbuch, Oren Ziv, Boris Brant, Evan Elliot, Shachaf Gelberg, Moran Titelbaum, Omry Koren, Orly Avni

**Affiliations:** a Azrieli Faculty of Medicine, Bar Ilan University, Safed, Israel; University of California, San Diego

**Keywords:** microbiome, autoimmunity, stress

## Abstract

Stressful life events are considered a risk factor for autoimmune disorders, though the mechanisms are unclear. Here we demonstrate that chronic social stress induces virulence-associated transcriptional patterns in the murine gut microbiota. The stress-influenced microbiota increased the presence of effector T helper cells in the mesenteric lymph nodes, including myelin-autoreactive cells. Inhibition of the bacterial quorum sensor QseC, which is also responsive to norepinephrine, diminished the presence of effector T helper cells and bacteria such as Acinetobacter in the mesenteric lymph nodes, without remarkably affecting the gut microbial composition. Together, our results delineate a model in which the immune reaction to stress-responsive microbiota may compromise tolerance to self and therefore may increase the risk for autoimmune diseases in susceptible individuals.

**IMPORTANCE** How do stressful life events increase the risk for autoimmune disorders? Here we show that chronic social stress in mice promotes the expression of virulent genes in the gut microbiota and alters the microbial translocation into the mesenteric lymph nodes. Our results also suggest that the consequent immune response to the stress-affected microbiota may endanger the tolerance for self. The presence of specific translocated bacteria and the immune response in the mesenteric lymph nodes can be diminished using an inhibitor of the bacterial communication system without drastically affecting the gut microbial composition as antibiotics do.

## Abstract (precis)

How do stressful life events increase the risk for autoimmune disorders? Here we show that chronic social stress in mice promotes the expression of virulent genes in the gut microbiota and alters the microbial translocation into the mesenteric lymph nodes. Our results also suggest that the consequent immune response to the stress-affected microbiota may endanger the tolerance for self. The presence of specific translocated bacteria and the immune response in the mesenteric lymph nodes can be diminished using an inhibitor of the bacterial communication system without drastically affecting the gut microbial composition as antibiotics do.

## INTRODUCTION

The incidence of autoimmune diseases is estimated at 5% worldwide (1). Although the etiology has a genetic element, the concordance rates in monozygotic twins are significantly below 50% for many autoimmune diseases, indicating a considerable influence of the environment. Altered microbial composition was found in humans with autoimmune diseases, and the causative role of the microbiota in the development of autoimmunity was demonstrated in several murine models (2–9).

Many studies support the notion that stressful life events play a role in the etiopathogenesis of autoimmune disorders (10, 11). Stress-triggered neuroendocrine hormones lead to immune dysregulation (12–14), but considering the recently appreciated gut-brain-microbiota axis (15) and the well-known microbiota-immune interactions (16–19), we asked whether and how the brain-microbiota-immune triangle is involved in stress-promoting autoimmunity.

## RESULTS

### Social defeat promotes virulent microbiota phenotype.

**FIG 1:**
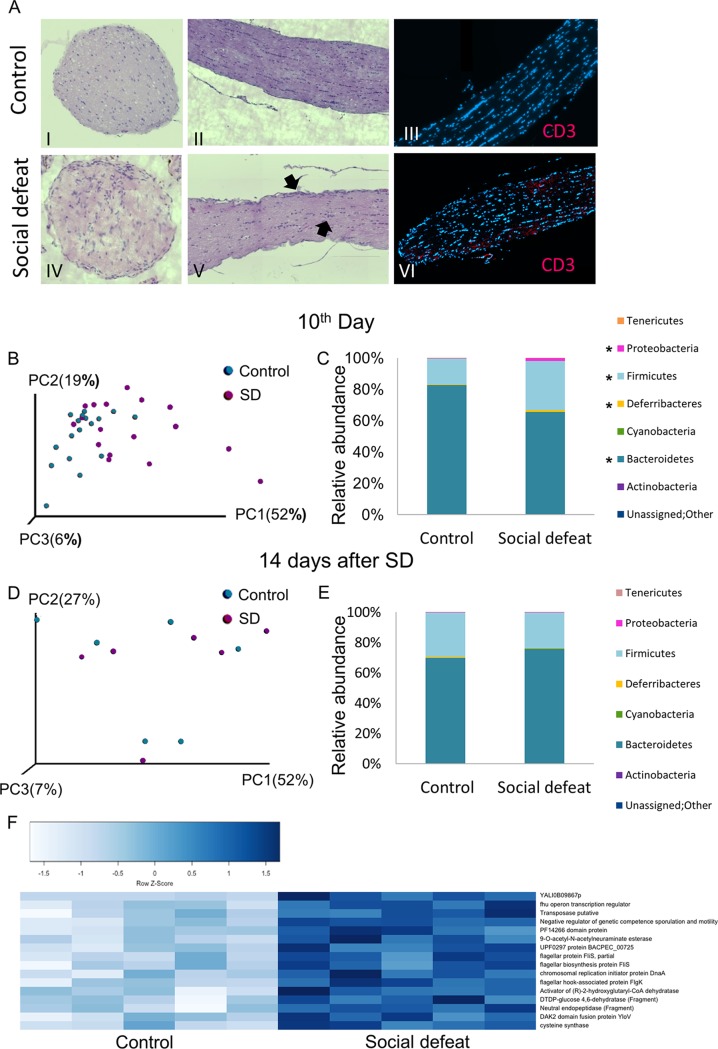
Social defeat (SD) promotes virulent microbiota phenotype. (A) Histological analysis (hematoxylin and eosin) of optic nerves sections and immunostaining (4′,6-diamidino-2-phenylindole [DAPI], blue; anti-CD3ε, red) from control (I to III) and SD (IV to VI) 2D2 mice. (B) PCoA plots of weighted UniFrac distances at the end of the SD session (10th day) for fecal microbiotas from control and SD group C57BL/6 mice (*n* = 17 from 3 independent experiments). *, *P* value < 0.05. (C) Taxonomical plots for the samples in panel B *, *P* value < 0.05. (D) PCoA plots of weighted UniFrac distances 14 days after the end of the SD session for fecal microbiotas from control and SD group C57BL/6 mice. (E) Taxonomical plots for the samples in panel D. (F) Heat map presenting genes more abundant in SD group C57BL/6 mice in comparison to control, identified by metatranscriptomic analysis of cecal contents (*n* = 5 each; *P* value < 0.001). The full list is in Fig. S1J; see also Fig. S1 as a whole.

To determine whether social stress increases the risk for autoimmunity in genetically susceptible mice, myelin oligodendrocyte glycoprotein (MOG)-specific T cell receptor transgenic (2D2) mice, of which 30% develop spontaneous autoimmune optic neuritis within their first year of life (20), were subjected to chronic social defeat (SD). SD is an established model for social stress and depression, wherein an intruder is repeatedly exposed to attacks and threats from a dominant resident (Fig. S1A) (21). Indeed, 5/6 (83%) 2D2 mice developed optic neuritis after 5 weeks of SD sessions, compared to 2/6 (33%) of the controls (Fig. 1A).

10.1128/mSystems.00292-18.1 FIG S1 (A) Photograph showing social defeat interaction between an ICR retired male breeder mouse (white) and C57BL/6 young male mice (black). (B to D) Microbial abundance plots of feces from SD group and control mice on the 10th day of the SD session for *Bacteroidetes* (B), *Firmicutes* (C), and *Proteobacteria* (D). (E) Quantitative PCR using *Gammaproteobacteria*-specific primers for feces of SD and control mice on the 3rd and 10th days of the SD session. (F) α-Diversity (Faith’s phylogenetic diversity) of fecal samples from SD and control mice on the 10th day of the SD session. (**G**) Mouse weights on days 0 and 10 of the SD session and 2 weeks after the SD session. (H) PCoA plot of weighted UniFrac distances at the end of the SD session, including two pools of three ICR aggressor mice each. (I) Predictive metagenomic analysis (PICRUSt) followed by LEfSe analysis showing differential pathways in control versus socially defeated mice; graph presents only pathways higher in SD group mice (linear discriminant analysis score > 3). CPS, cellular process and signaling; GIP, genetic information processing. (J) List of RNA sequences that were expressed at higher levels in cecal SD. Error bars represent standard deviations. Download FIG S1, PDF file, 0.5 MB. Copyright © 2019 Werbner et al. 2019 Werbner et al. This content is distributed under the terms of the Creative Commons Attribution 4.0 International license.

To explore the involvement of the microbiota in the induction of autoimmunity after SD, we chose to use wild-type (WT) C57BL/6 mice. 16S rRNA gene sequences of feces from control mice and mice subjected to 10 days (1 session) of SD were significantly separated using weighted UniFrac (*P* value = 0.02) (Fig. 1B). Phylum profiling showed that the SD group had a significantly higher *Firmicutes*-to-*Bacteroidetes* ratio than the control (false-discovery rate [FDR] < 0.05), as previously demonstrated in similar model systems and in many cases of disease-associated dysbiosis (22–24), and an increased relative abundance of *Deferribacteres* and *Proteobacteria* (FDR = 0.004 and FDR = 0.0004) (Fig. 1C). At the genus and species levels, significant enrichment in the SD group feces included *Oscillospira*, *Ruminococcus* (FDR = 0.003), and *Dehalobacterium* (*P* value = 0.028) (*Firmicutes*, 2- to 3-fold), Mucispirillum schaedleri (FDR = 0.004) (*Deferribacteres*, ∼3-fold), and *Bilophila* (FDR = 0.0004) (*Proteobacteria*, ∼6-fold) (Fig. S1B to E and Table S1). *Bilophila* and *Dehalobacterium* were recently demonstrated to be more abundant in the gut of human patients with multiple sclerosis (MS) than in controls (4, 25). α-Diversity between SD group fecal microbiota and the control microbiota did not reach statistical significance (*P* value = 0.238) (Fig. S1F). The microbial shift probably cannot be explained by decreased consumption of food, since the control group had a similar average weight (Fig. S1G). A weighted UniFrac principal-coordinate analysis (PCoA) plot suggested that the microbial profile of the cohousing aggressor mice at the end of the SD session was more similar to the control microbial profile than to the SD profile (Fig. S1H). Fourteen days after the last exposure of the mice to the aggressor, the structure of the SD-associated bacterial community nearly returned to homeostasis without any significant change (Fig. 1D and E).

10.1128/mSystems.00292-18.4 TABLE S1 Alterations in the relative abundances of bacteria in the feces of SD group mice on the 10th day of the SD session in comparison to the control. See also Fig. 1. Download Table S1, PDF file, 0.06 MB. Copyright © 2019 Werbner et al. 2019 Werbner et al. This content is distributed under the terms of the Creative Commons Attribution 4.0 International license.

Alterations in the transcriptional patterns of the stress-responsive bacteria may have a crucial impact on pathogenicity. A predictive metagenomics (PICRUSt) analysis (26) estimated that the SD metagenome was enriched in bacterial functions related to virulence traits (Fig. S1I). To assess the actual changes in transcriptional patterns, we performed cecal metatranscriptomics analysis (Fig. 1F). As predicted, virulence-associated transcriptional patterns were dominant, but more specifically, they comprised genes such as the *fhu* (ferric uptake) operon transcription regulator, which involves diverse functions ranging from iron homeostasis to regulation of virulence (27); the chromosomal replication initiator protein DnaA (28), which is required for DNA synthesis; transposase, which may play a role as a bacterial mutagenic agent (29); the flagellar biosynthesis protein FliS and flagellar hook-associated protein FlgK, facilitating motility and host colonization; and pathways associated with biosynthesis and growth, including DTDP-glucose 4,6-dehytratase, involved in the synthesis of the cell wall (30), and 9-*O*-acetyl-*N*-acetylneuraminate esterase, required for growth on sialic acid, which functions as a nutritional source and surface decoration and cell signaling in host-microbe interactions (31). Together, these data demonstrate that the microbiota’s response to stress encompasses alterations in the composition and elevation in transcriptional patterns associated with growth, motility, and host-pathogen communication.

### Alterations in microbiota compositions and increased T helper (Th) effector response in SD group MLNs.

**FIG 2:**
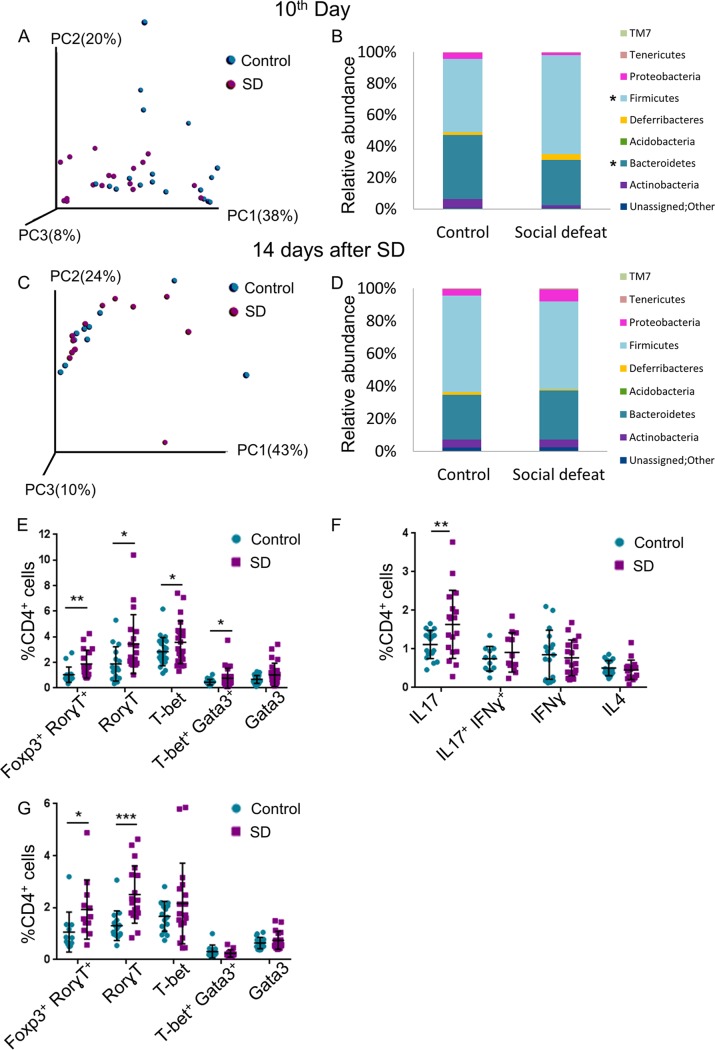
Alterations in microbiota compositions and increased Th effector response in SD group MLNs. (A) PCoA of weighted UniFrac distances at the end of the SD session (10th day) for microbiotas for MLNs from control and SD group C57BL/6 mice. (B) Abundance plots of the samples in panel A (panels A and B summarize three independent experiments; *n* = 18). (C) PCoA of weighted UniFrac distances 14 days after the end of the SD session for microbiotas for MLNs from control and SD group C57BL/6 mice. (D) Abundance plots of the samples in panel C (panels C and D summarize two independent experiments; *n* = 12). (E) Flow cytometry with anti-lineage-specifying transcription factor antibodies, as indicated, at the end of the SD session (10th day). (F) Flow cytometry with anti-cytokine antibodies, as indicated, at the end of the SD session (10th day). (G) As in panel E, 14 days after the end of the SD session. For panels E and F, *n* =16 to 23 from 4 independent experiments; for panel G, *n* = 12 to 18 from 2 or 3 independent experiments. Asterisks indicate significant differences between control and SD using a one-tailed *t* test, as follows: *, *P* value < 0.05; **, *P* value < 0.01; and ***, *P* value < 0.001. Error bars show standard deviations; see also Fig. S2.

Motile virulent bacteria may acquire the ability to cross the epithelial barrier. We therefore explored the SD-inducible changes in the bacterial composition in the mesenteric lymph nodes (MLNs), which drain the gut lymph. At the end of the SD session, the microbial clustering of the SD group and control MLNs were partially separated (Fig. 2A), and as in the gut, the *Firmicutes*/*Bacteroidetes* ratio was significantly higher in the SD group MLNs (FDR < 0.005) (Fig. 2B and Table S2). Genera/species that were significantly enriched in at least one of the experiments included *Adlercreutzia* (*P* value = 0.0259; *Actinobacteria*), Brevundimonas diminuta (*P* value = 0.044; *Alphaproteobacteria*; an opportunistic pathogen in immunocompromised hosts [32]), and *Helicobacter* (*P* value = 0.022; *Epsilonproteobacteria*; highly prevalent pathobiont that can lead to gastritis, peptic ulcer disease, and possibly autoimmune diseases [33]), whereas genera/species that were relatively less abundant in SD group MLNs included *Sutterella* (*P* value = 0.0163; *Betaproteobacteria*; reduced in the gut of human patients with MS) and *Prevotella* (*P* value = 0.010; *Bacteroidetes*; either reduced or increased in the gut of MS patients [34], probably since different species have different functions [35]) (Fig. 2B and Table S2). Fourteen days later, the clustering of the microbial composition in the SD group MLNs was indistinguishable from that of the control MLNs (*P* value = 0.717) (Fig. 2C); however, the enrichment in *Adlercreutzia* (*P* value = 0.009), Brevundimonas diminuta (*P* value = 0.021), and *Helicobacter* (*P* value = 0.007) was maintained (Fig. 2D and Table S2). *Dehalobacterium* (as mentioned above, higher in MS patients [36]) was also increased (*P* value = 0.006). These changes may suggest that the SD group MLNs harbor translocated pathobionts, symbionts with a pathological potential.

10.1128/mSystems.00292-18.5 TABLE S2 (A) Alterations in the relative abundance of bacteria in the feces of SD group mice on the 10th day of the SD session in comparison to the control. (B) Alterations in the relative abundances of bacteria in the feces of SD group mice 14 days after the end of the SD session in comparison to the control. See also Fig. 2. Download Table S2, PDF file, 0.07 MB. Copyright © 2019 Werbner et al. 2019 Werbner et al. This content is distributed under the terms of the Creative Commons Attribution 4.0 International license.

10.1128/mSystems.00292-18.2 FIG S2 (A) Schematic illustration of T helper cell differentiation. (B) Flow cytometry with anti-lineage-specifying transcription factors antibodies, as indicated, after 3 days of SD sessions from control MLNs and MLNs from SD group C57BL/6 mice. (C) Flow cytometry with anti-lineage-specifying transcription factors antibodies, as indicated, after 10 days of SD sessions from control MLNs and MLNs from SD group C57BL/6 mice. (D) Flow cytometry with anti-lineage-specifying transcription factors antibodies, as indicated, 2 weeks after the last SD session, from control MLNs and MLNs from SD group C57BL/6 mice. (E) Representative flow cytometry analysis of one experiment 2 weeks after SD sessions. MLN cells were stained for CD4, Foxp3, and Rorγt. Error bars represent mean standard deviations. Download FIG S2, PDF file, 0.8 MB. Copyright © 2019 Werbner et al. 2019 Werbner et al. This content is distributed under the terms of the Creative Commons Attribution 4.0 International license.

The immune system has the challenging task of maintaining tolerance to commensal and beneficial bacteria while simultaneously remaining competent to launch an effective immune response against insults from pathobionts and external pathogens (1, 16, 19, 37). T helper (CD4^+^) cells have a fundamental role in that challenge: their first interaction with the antigen promotes their differentiation into either regulatory (Treg) or effector lineages, mainly Th1, Th2, and Th17, defined by expression of the lineage specifying transcription factors Foxp3, T-bet, Gata3, and Rorγt, respectively (Fig. S2A). The Th1, Th2, and Th17 effector cells differentially express distinct set of hallmark cytokines—gamma interferon (IFN-γ), interleukin 4 (IL-4), and IL-17, respectively—that eventually instruct the strategy of the immune response. Treg cells play a pivotal role in dampening an inappropriate immune response against self-antigens or commensal bacteria. The differentiation process of Th cells presents a certain degree of plasticity, and under specific circumstances, Th cells can gain the expression of the opposing cytokines or even redifferentiate toward a different Th lineage. The gut in particular uses this Th cell flexibility to maintain a fine balance of tolerance versus inflammation. In light of their strategic position in the immune response, when their function is dysregulated, Th cells may initiate or promote a variety of autoimmune diseases; Th1 and Th17 responses dominate many autoimmune diseases, including MS (38, 39). To assess potential diversion in Th cell differentiation in response to stress, MLN cell suspensions were analyzed by flow cytometry using antibodies specific to the Th lineage-specifying transcription factors. After 3 days of SD, no significant change in the composition of Th subtypes at the MLNs was observed (*P* value = 0.17 for RorγT and *P* value = 0.35 for Foxp3) (Fig. S2B); however, on the 10th day, at the end of the SD session, a modest but significant increase in the percentage of the effector cells expressing RorγT, T-bet, and T-bet/Gata3 appeared in the SD group MLNs (in 83% [*P* value = 0.01], 26% [*P* value = 0.03], and 70% [*P* value = 0.042], respectively) (Fig. 2E). The percentage of Treg cells was almost unchanged (*P* value = 0.78) (Fig. S2C); however, the percentage of Foxp3/RorγT-expressing cells increased (by 81%, *P* value = 0.004) (Fig. 2E). We currently cannot distinguish whether these populations represent either Ex-Treg or Ex-Th17 cells (that were evolved from either Foxp3^+^ or RorgT^+^ Th cells, respectively) or, alternatively, a distinct lineage (40–44). Accordingly, the abundance of IL-17-expressing Th cells was higher in SD group MLNs than control MLNs (in 47% [*P* value = 0.015] (Fig. 2F). A significant increase in the frequency of Foxp3/RorγT- and RorγT-expressing cells was also observed 14 days after the end of the SD session (in 81% [*P* value = 0.023] and 92% [*P* value = 0.0002], respectively) (Fig. 2G). The relative abundance of FoxP3-expressing cells was slightly increased at this stage (in 5.5% [*P* value = 0.028]) (Fig. S2D) and may reflect an attempt to resolve the immune response. In summary, SD group MLNs harbor a higher percentage of effector Th cells even after the gut microbial composition returned to homeostasis. The modest increase in the relative abundance of effector Th cells in the SD group MLNs may suggest stimulation of only selected clones in response to stress.

### Effects of social stress on immune regulation are mediated by the gut microbiota.

**FIG 3:**
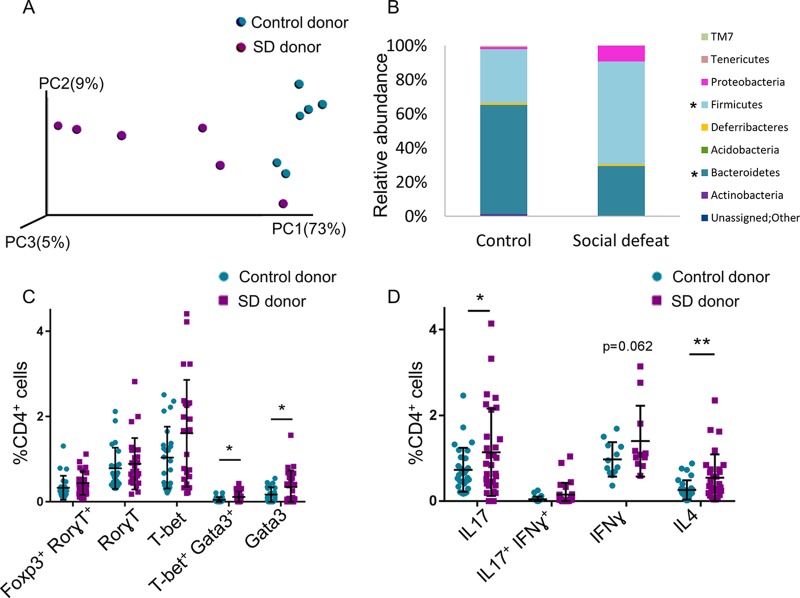
Effects of social stress on immune regulation are imprinted on the gut microbiota. (A and B) PCoA plot of weighted UniFrac distances (*P* value = 0.001) (A) and taxon summary plot of bacterial populations at the MLNs of GF Swiss Webster mice (B) 12 days after fecal transplantation from control and SD group C57BL/6 mice. (C and D) Flow cytometry with anti-lineage-specifying transcription factors (C) and cytokine antibodies (D), as indicated, 12 days after fecal transplantation from control and SD group C57BL/6 mice. For panel C, *n* = 24 from 4 independent experiments; for panel D, *n* = 11 to 24 from 2 to 4 independent experiments. Means were significantly different using one-tailed *t* test as follows: *, *P* value < 0.05; **, *P* value < 0.01; and ***, *P* value < 0.001. Error bars represent standard deviations; see also Table S3.

Since the immune system engages in a bidirectional cross talk with both the microbiota and neuroendocrine system, the increased abundance of effector Th cells in the SD group MLNs may indicate direct effects of the stress hormones, may occur indirectly through a stress-responsive microbiota, or may represent a combination of both. To determine the effect of the SD-associated microbiota on Th cell differentiation in the MLNs, feces of SD group and control mice were transplanted into germfree (GF) male Swiss Webster mice. Twelve days later, the microbial compositions in the MLNs differed (Fig. 3A); MLNs of mice receiving feces from SD group mice presented a significantly higher *Firmicutes*/*Bacteroidetes* ratio than the control; the relative abundance of the species Lactococcus garvieae (*P* value = 0.007; *Firmicutes*; an emerging human pathogen [45]) and *Proteus* (*P* value = 0.002; *Gammaproteobacteria*; associated with rheumatoid arthritis [46]) were significantly higher in the MLNs of the GF mice receiving feces from SD group mice (from 0% to 8.5% and 0.2%, respectively (Fig. 3B and Table S3).

10.1128/mSystems.00292-18.6 TABLE S3 Alterations in the relative abundances of bacteria in the MLNs of germfree mice 12 days after fecal transplantation from SD group mice in comparison to the control. See also Fig. 3. Download Table S3, PDF file, 0.05 MB. Copyright © 2019 Werbner et al. 2019 Werbner et al. This content is distributed under the terms of the Creative Commons Attribution 4.0 International license.

MLNs of GF mice receiving feces from SD group mice exhibited a higher percentage of effector Th cells expressing T-bet, T-bet/Gata3, and Gata3 than the MLNs of the control recipient mice (in 55% [*P* value = 0.029], 140% [*P* value = 0.013], and 100% [*P* value = 0.022], respectively) (Fig. 3C) and a higher percentage of cells expressing IL-17 (*P* value = 0.027), IFN-γ (did not reach statistical significance [*P* value = 0.062]), and IL-4 (*P* value = 0.005) (in 56%, 44%, and 110%, respectively) (Fig. 3D). The increase in Gata3/IL-4-expressing Th cells is most likely a result of the Th2 bias of the GF mice (16). The transferable MLN phenotype by the SD-selected microbiota demonstrates that the ability to divert the differentiation process of the effector Th cells in the MLNs is an intrinsic feature of the altered microbiota, although we cannot rule out a cooperative effect of the neuroendocrine system.

### Microbiota-dependent autoreactive response of the SD group MLNs.

**FIG 4:**
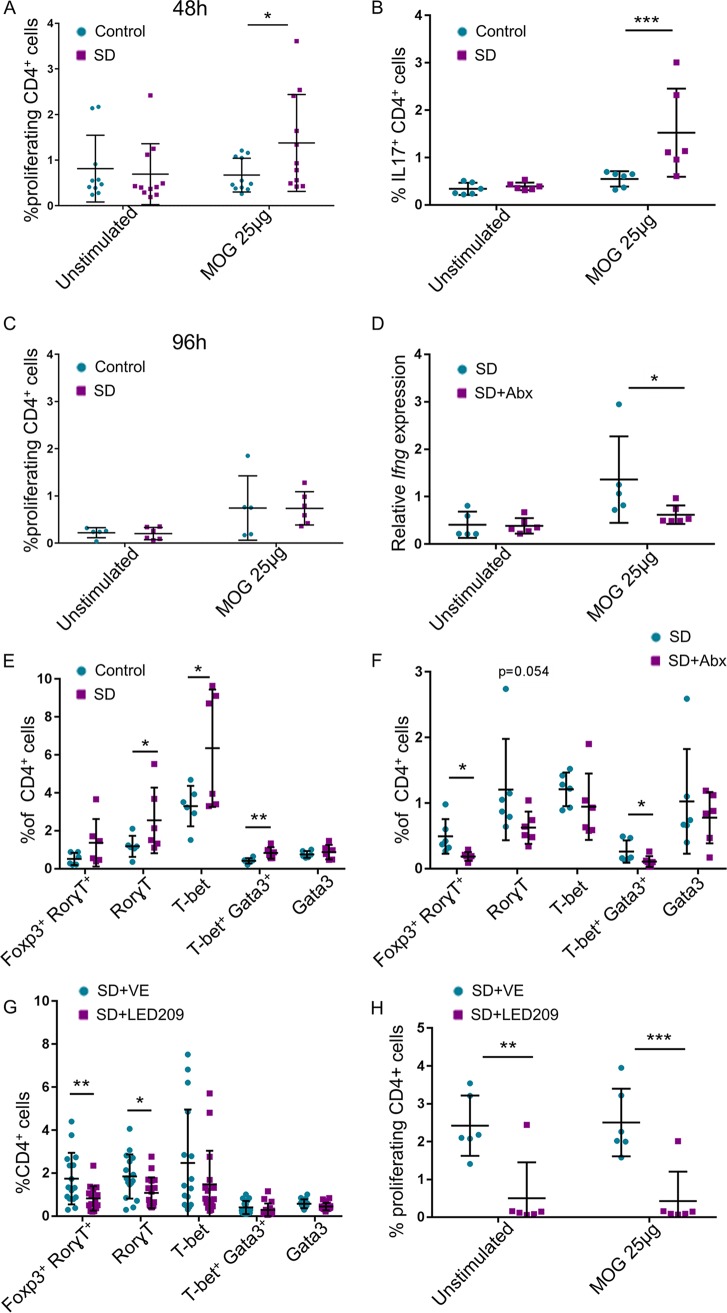
Microbiota-dependent autoreactive response of the SD group MLNs. (A) MLN cells at the 10th day of SD were seeded with or without MOG_35-55_ peptide for 48 h, and the proliferation rate of CD4^+^ cells was measured. (B) Flow cytometry of unstimulated cells and cells stimulated for 48 h with MOG_35-55_ using anti-IL-17 antibodies. (C) Same as in panel A, but cells were stimulated for 96 h. For panel A, *n* = 11 from 2 independent experiments; for panel B, *n* = 6; and for panel C, *n* = 6. (D) SD session was done with or without broad-spectrum antibiotics (Abx), and on the 10th day MLN cells were seeded for 4 h with or without MOG_35-55_ stimulation for assessing the expression of *Ifng* (*n* = 6). We did not observe differences in the expression level of *Il17*, possibly due to the short time of stimulation. (E) Flow cytometry with anti-lineage-specifying transcription factors antibodies, as indicated, of 2D2 SD group MLN and control 2D2 MLN cells at the end of the 5th week, as in Fig. 1A. (F) Flow cytometry with anti-lineage-specifying transcription factors antibodies, as indicated, of MLN cells from 2D2 SD group and antibiotic-treated 2D2 SD group mice. (G) Mice were treated with LED209 or a control vehicle, and 14 days after the end of the SD session, flow cytometry with anti-lineage-specifying transcription factors antibodies, as indicated, was performed on the MLN cells. (H) Fourteen days after the end of SD session, MLN cells as in panel G were seeded with or without MOG_35-55_ peptide for 48 h and the proliferation of CD4^+^ cells was measured. Means were significantly different using one-tailed *t* test as follows: *, *P* value < 0.05; **, *P* value < 0.01; and ***, *P* value < 0.001. Error bars represent standard deviations; see also Fig. S3.

MS is a chronic inflammatory demyelinating disease of the central nervous system caused by myelin-specific, self-reactive T lymphocytes. T cells with high affinity for central nervous system (CNS) autoantigens are present in healthy individuals, but they normally remain quiescent unless they are activated in the periphery. To determine whether the stress-induced Th cell response possesses self-reactivity, and therefore may increase the risk for autoimmune disease such as MS, we assessed, as a proof of concept, the response of MLN-derived Th cells of C57BL/6 mice to the myelin peptide MOG_35-55_. Indeed, SD group MLNs exhibited a higher percentage of proliferating Th cells after 48 h of *in vitro* stimulation with MOG than control MLNs (Fig. 4A), as well as an increased percentage of IL-17-expressing Th cells (Fig. 4B). Stimulation with MOG for 96 h induced proliferation of Th cells in control MLNs as well, probably as a result of a first stimulation of naive Th cells *in vitro*, as the autoreactive potential exists in healthy mice (Fig. 4C). To determine the role of the microbiota in the SD-inducible autoreactive response to MOG, we treated C57BL/6 mice with broad-spectrum antibiotics 2 weeks before and during the SD session. We assessed the level of expression of *Ifng* mRNA by MLN-derived Th cells after only 4 h of *in vitro* stimulation with MOG to clearly distinguish between first *in vivo* to *in vitro* stimulation of naive Th cells. The expression of *Ifng* by SD group MLN-derived Th cells was, as expected, higher than the control in response to MOG; however, this response was abrogated if the mice were treated with antibiotics (Fig. 4D). Similarly, MLNs of 2D2 mice, which express a MOG-specific T cell receptor (TCR) (Fig. 1A), possessed a higher percentage of effector cells expressing Rorγt and T-bet at the end of the 5th week of the SD sessions (Fig. 4E). The percentage of the effector cells was reduced if the mice were treated with antibiotics before and during the SD procedure (Fig. 4F). Thus, together, these results demonstrate that SD group MLNs harbor microbiota-dependent self-reactive experienced Th cells.

Major mediators of the stress response are the stress hormones, including epinephrine and norepinephrine (12, 47). Several species of *Proteobacteria* are known to be epinephrine/norepinephrine responsive through their quorum sensor C (QseC) receptor (48). Activation of this sensor has the potential to affect growth and virulence. A database search (PubMed) revealed that many additional *Proteobacteria* (and few members of other phyla) possess QseC as well. Epinephrine/norepinephrine can also affect the growth and virulence of some anaerobic species (48, 49), although the underlying mechanisms are yet unknown. Daily treatment with the organic QseC inhibitor LED209 (50), just before the SD session, reduced the percentage of effector Th cells expressing Rorγt in the MLNs, as assessed 14 days after the end of the SD session, in comparison to their presence in the MLNs of vehicle-treated SD group mice (Fig. 4G). Interestingly, even though the effect of LED209 on the microbial composition was minor, the relative abundance of Acinetobacter (*Gammaproteobacteria*; from 0.75% to 0% [*P* value = 0.0218]) in one experiment, and of Propionibacterium acnes (*Actinobacteria*; from 0.26% to 0.016% [*P* value = 0.0131]) in another one, was significantly decreased in the MLNs of LED209-treated mice (Fig. S3A to F and Table S4). Both bacteria were shown *in vitro* to be epinephrine/norepinephrine responsive. Acinetobacter, an opportunistic multidrug-resistant human pathogen (51), was enriched in the gut of MS patients (4) and suggested as an etiologic cause of MS (52, 53). P. acnes is a dominant member of the skin microbiota but is also able to invade and persist in epithelial cells and circulating macrophages to subsequently induce sarcoidosis, low-grade inflammation, and metastatic cell growth in the prostate gland and infiltrate the brain parenchyma, contributing to pathogenic processes in neurodegenerative disorders (54). Concomitant with the microbial changes, the proliferation rate *in vitro* of Th cells derived from SD group MLNs was diminished if the mice were treated with LED209 (Fig. 4H). The results were similar with and without the presence of MOG peptide, possibly since the assay was done 14 days after the end of the SD session, when the antigen was already found in sufficient amounts in the MLNs. Together, these results demonstrate that specific targeting of bacterial stress sensing machinery decreases the presence of potential pathogenic bacteria in the lymph nodes, and consequently the immune response, without drastically affecting the microbial composition.

10.1128/mSystems.00292-18.3 FIG S3 (A and B) PCoA plot of weighted UniFrac distances (A) and taxon summary plot of stool bacterial populations (B) at the end of SD session that include gavage with vehicle or LED209. (C and D) As in panels A and B 2 weeks after the SD session. (E and F) PCoA plot of MLNs (E) and taxon summary plots (F) as in panels C and D 14 days after the SD session. Download FIG S3, PDF file, 0.1 MB. Copyright © 2019 Werbner et al. 2019 Werbner et al. This content is distributed under the terms of the Creative Commons Attribution 4.0 International license.

10.1128/mSystems.00292-18.7 TABLE S4 (A) Alterations in the relative abundances of bacteria in the feces of LED209-treated SD group mice in comparison to the control of vehicle-treated mice, 14 days after the end of the SD session. (B) Alterations in the relative abundances of bacteria in the MLNs of LED209-treated SD group mice in comparison to the control of vehicle-treated mice 14 days after the end of the SD session. See also Fig. 4. Download Table S4, PDF file, 0.06 MB. Copyright © 2019 Werbner et al. 2019 Werbner et al. This content is distributed under the terms of the Creative Commons Attribution 4.0 International license.

## DISCUSSION

We show that an environmental trigger such as social stress affects the bacterial composition and transcriptional patterns in a way that enforces an immune response with potential deleterious consequences to self-tolerance. This might be a hit-and-run effect since although the gut microbial community recovered after the cessation of the stress, the microbial alterations and the immune response in the MLNs persisted, highlighting the consequences of an early stress-inducible disturbance on the homeostasis later in life. In that aspect, many of the stress-responsive bacteria that we found are known to be associated with autoimmunity and other diseases.

The metatranscriptomics analysis demonstrated probably the ability of microbial communities to gauge and consequently to adapt their transcriptional patterns to chronic social stress response, which may reflect a fragile fitness of their host. The stress-associated virulent phenotype can explain, for example, how under healthy conditions, the relative abundance of *Proteobacteria* in the human gut can transiently increase from 2.5% to 45% without clinical signs, whereas under certain undefined circumstances, they do trigger inflammatory responses (55–57). The stress-inducible transposase expression may facilitate increased genetic diversity and horizontal gene exchange, which is high in some proteobacterial strains and might contribute to their fitness advantage in unstable environments (55, 58). Therefore, gut bacterial composition *per se* does not necessarily reflect the threat to health, and the bacterial transcriptional patterns should be considered as well.

The number of bacterial species in the mammalian gut is estimated to be more than 1,000, and the number of their genes in the collective microbiome is more than 150-fold greater than in the genome of their host (55, 59). Moreover, microorganisms in the human gut encompass archaeal, viral, and fungal species as well, making the human intestine a rich source of antigens with potential for cross-reactivity and immunomodulation. We currently cannot distinguish whether the microbiota-induced self-reactivity is a result of molecular mimicry, bystander activation, or combination of both (60). Further exploration of this interkingdom interaction may facilitate the development of tailored interventions in stress-associated illnesses such as autoimmune disorders and depression. However, the Holy Grail, a specific causative agent for distinct disease, can be elusive; most likely, many bacteria have redundant environmental sensors, structural features, and functions that may explain the current difficulty in identifying distinguished microbial signature for specific diseases, such as MS (4, 5, 34, 36, 61–66). But while different bacteria may initiate similar outcomes, our results in repetitive experiments and even in the same experiment with seemingly very similar genetics and environmental factors show that a specific environmental factor, such as stress, can induce a variety of microbial compositions with potential for alternative outcomes. This may suggest that microenvironmental factors and even local stochastic events also play a role in promoting a threat out of the wide range of potential destructive consequences of chronic stress.

## MATERIALS AND METHODS

### Animal model.

ICR retired male breeder mice (8 months or older) and C57BL/6 male mice, aged 8 to 10 weeks (Harlan), were housed under specific-pathogen-free (SPF) conditions (5 per cage) and were allowed to habituate to the animal facility for at least 5 days prior to testing. The cages were maintained in a 12:12 h light-dark schedule with lights on from 6:00 to 18:00. Food and water were available *ad libitum*.

All experimental procedures were approved by the Bar Ilan University ethics committee (approval numbers 16-03, 47-07, and 53-06) following IACUC guidelines. GF Swiss Webster male mice were maintained in the Bar-Ilan GF facility and GF status was confirmed before fecal microbiota transplantation (FMT). After FMT, mice were maintained in a conventional mouse facility. Myelin oligodendrocyte glycoprotein (MOG)-specific T cell receptor transgenic mouse (2D2) breeding pairs were obtained from The Jackson Laboratory, and the colony was established in-house using mixed pairs of mutant and WT mice; genotyping was done according to The Jackson Laboratory recommendations.

### SD protocol.

According to the chronic social defeat (SD) model (21, 67), an intruder is repeatedly exposed to attacks and threats from a dominant aggressive resident (Fig. S1A). For a session of 10 consecutive days, a young C57BL/6 intruder mouse was physically exposed to a resident, dominant retired breeder ICR mouse for 5 min a day, and subsequently the mice were housed in the same cage across a transparent divider for the next 24 h to enable transmission of sensual cues while preventing any physical contact. Wounding was monitored throughout the SD cycle. Only mice with wounds limited to superficial injury were included in analysis. Control mice were housed in divided cages with one mouse in each side. As for the 2D2 experiments, the young 2D2 mice were exposed to 5 rounds of short sessions of 5 days each with 2 resting days in between.

### Wide-range antibiotic administration.

For C57BL/6 mice, a combination of 1.22 g/liter of metronidazole (Acros Organics), 0.22 g/liter of ciprofloxacin (Sigma-Adrich), and 0.725 g/liter of vancomycin hydrochloride (Goldbio) was dissolved in the drinking water and administered 2 weeks before SD and during the 10 days of the SD session. For 2D2 mice, sulfamethoxazole (MP Biomedicals) and trimethoprim (Cayman Chemical) (68), 80 mg/kg of body weight per day (0.25 mg/ml), were dissolved in the drinking water and were administered 2 weeks before the experiment and during the 5 weeks of SD sessions.

### LED209 administration.

LED209 (Cayman Chemical) administration was done by oral gavage of 20 mg/kg of body weight in 10% dimethyl sulfoxide (DMSO; Sigma-Aldrich), 10% Kolliphor EL (Sigma-Aldrich), 30% polyethylene glycol 400 (PEG 400; Sigma-Aldrich), and 50% carbonate buffer (pH 10) 30 min before SD (7). The control was done by oral gavage with the same solution (vehicle) except LED209.

### Quantitative real-time PCR of stool and lymph node samples.

DNA was extracted from stool samples using the Mobio PowerSoil DNA (MoBio) extraction kit. Quantitative PCR was performed in sealed 96-well plates using a StepOnePlus PCR system (Applied Biosystems) or in 384-well plates using ViiA7 (Thermo Fisher Scientific). Each reaction included Fast SYBR green master mix (Applied Biosystems), class-specific primer pairs at 0.2 μM, and 25 ng of DNA template in a final volume of 10 μl or *Ifng* primers at 0.2 μM and 10 ng of cDNA template. The following PCR protocol was used: 95°C (30 s) and then 95°C (5 s), 60°C (15 s), and 72°C (10 s) for 40 cycles. Each sample was run in triplicate, and the mean threshold cycle (*C_T_*) value was used to calculate delta *C_T_* values using the glyceraldehyde-3-phosphate dehydrogenase (GAPDH) housekeeping gene or total 16S rRNA. Values were then normalized to average control *C_T_* values to calculate delta delta *C_T_*. *P* values were calculated using the one-tailed Student *t* test. The following primers were used: gammaproteobacterial primers 1080F (TCGTCAGCTCGTGTYGTGA) and 1202R (CGTAAGGGCCATGATG) (69), total bacterial primers F-TGGCTCAGGACGAACGCTGGCGGC and R-CCTACTGCTGCCTCCCGTAGGAGT (69), *Ifng* primers F-GCGTCATTGAATCACACCTG and R-TGAGCTCATTGAATGCTTGG, and GAPDH primers F-CTCCCACTCTTCCACCTTCG and R-CCACCACCCTGTTGCTGTAG.

### Bacterial 16S DNA sequencing.

Bacterial DNA was extracted from feces, cecal contents, and MLN using the Mobio PowerSoil DNA extraction kit (MoBio) following a 2-min bead beating step (Biospec). V4 or V2 of the 16S rRNA gene was amplified using PCR with barcoded primers (listed below) (70). DNA was then purified using AMPure XP magnetic beads (Beckman Coulter) and quantified using Quant-iT PicoGreen double-stranded DNA (dsDNA) assay (Thermo Fisher), and equal amounts of DNA were then pooled and sequenced. After sequencing on an Illumina MiSeq platform at the Faculty of Medicine Genomic Center (Bar Ilan University, Safed, Israel), reads were quality filtered and trimmed using Trimmomatic (71). Further analysis, including paired-end joining, demultiplexing, and chimera checking (using Usearch [72]), was performed; open-reference operational taxonomic unit (OTU) picking was done using QIIME (version 1.8.0) (73). OTUs with less than 0.01% of total reads were discarded. Multiple comparisons were accounted for using FDR. α-diversity was calculated using Faith’s phylogenetic diversity (PD) (74); β-diversity was calculated using the UniFrac distance metric (75). Nonparametric *P* values were calculated using 999 Monte Carlo permutations. Further analysis of predictive functional profiling was done using PICRUSt (26).

V2 primers were 27F (AATGATACGGCGACCACCGAGATCTACACGTACGTACGGTAGAGTTTGATCCTGGCTCAG) and 338R (CAAGCAGAAGACGGCATACGAGATGATGTATGTGGTCCACACTCATCATGCTGCCTCCCGTAGGAGT). Golay barcodes were added to reverse primers.

V4 primers were 515F (AATGATACGGCGACCACCGAGATCTACACGCTAGCCTTCGTCGCTATGGTAATTGTGTGYCAGCMGCCGCGGTAA) and 806R (CAAGCAGAAGACGGCATACGAGATAGTCAGTCAGCCGGACTACHVGGGTWTCTAAT). Golay barcodes were added to forward primers.

### Metatranscriptomics.

Cecal RNA was purified using the Mobio RNA purification kit (MoBio). Bacterial rRNA was depleted using Ribo-Zero (Epicentere). An RNA library was prepared using NEBNext Ultra Directional RNA library prep kit (New England BioLabs) for Illumina and sequenced using Illumina Hiseq (76), 100-bp single read, at the Technion Genome Center (Technion, Haifa, Israel). All rRNA sequences were removed using SortMeRNA (77), and all mouse RNA sequences were then removed using bowtie2 (78) with mm10 as a reference. The remaining sequences were analyzed using HUMAnN2 (79), and the resulting gene family file was used as input for a heatmap in R, showing only gene families with *P* values of <0.001. Reads were normalized to total numbers of reads that were annotated.

### Flow cytometry.

Cells were harvested from MLNs by mechanical dissociation and were taken for further analysis or seeded in primary medium containing Dulbecco modified Eagle medium (DMEM), 10% fetal bovine serum (FBS), 1% HEPES, 1% penicillin-streptomycin, 1% essential amino acids, 1% sodium pyruvate, 1% l-glutamate, 1% folic acid mix, 1% vitamins (all medium components were obtained from Biological Industries), and 0.01% β-mercaptoethanol (Sigma-Aldrich). Cells were stained for CD4 immediately after harvest for 20 min at room temperature (RT) and then stained for the different transcription factors using a Cytofix/Cytoperm kit (BD Biosciences) according to the manufacturer’s protocol. Cells were seeded for 4 h of stimulation with phorbol 12-myristate 13-acetate (PMA; 50 nM) and ionomycin (1 μM) (both from Sigma-Aldrich) in the presence of Golgi-stop and Golgi-plug reagents and then collected for cytokine staining using the BD Cytofix/Cytoperm kit (BD Biosciences). Proliferation was measured using Click-iT kit (Thermo Fisher Scientific) for flow cytometry. Cells were seeded for different periods as indicated in the figure legends, and 5 μM EDU (5-ethynyl-2′-deoxyuridine) was added for the last 4 h of the experiment. Cells were collected, and after extracellular staining for CD4^−^ Pacific blue (clone GK1.5), the manufacturer’s protocol was followed. All flow cytometry analyses were performed using a Gallios flow cytometer, and all analyses were performed using FlowJo software (BD Biosciences). Rorγt-phycoerythrin (PE) (clone AFKJS-9) and Gata3-eFluor 660 (clone TWAJ) antibodies were purchased from eBioscience; all other antibodies were purchased from BioLegend: Foxp3-Alexa Fluor 647 (clone 150D), T-bet–PE (clone 4B10), IFN-γ–Alexa Fluor 647 or 488 (clone XMG1.2), IL-17A–PE (clone TC11-18H10.1), and IL-4–Alexa Fluor 647 (clone 11B11).

### Fecal microbiota transplantation.

Fecal samples from socially defeated or control mice were suspended in 5 ml of sterile 1% phosphate-buffered saline (PBS; Biological Industries) immediately after sample collection. The suspension (200 μl per mouse) was administered into GF mice using gavage needles (each donor had one recipient). Twelve days after gavage, mice were sacrificed, and MLNs were collected for further analysis.

### Histology.

Tissue was fixed using 4% formaldehyde (Sigma-Aldrich) for at least 24 h; after fixation, tissues were paraffin embedded (Hista-Flex Ultra; Poth-Hille & Co.). Sections of 6 μm were stained with hematoxylin and eosin (American Mastertech Scientific Inc.) and were analyzed using AXIO scan Z1 (Zeiss). For immunofluorescent dyes, after rehydration of slides, samples were incubated for 1 h with anti-CD3ɛ-Alexa Fluor 647 (clone 145-2C11; Biolegend) in 1% donkey serum (MP Biomedicals).

### Statistical analyses.

Statistical analyses of differences between groups were performed with Prism 7.0 software (GraphPad) using unpaired, one-tailed Student *t* test or by Microsoft Excel. For 16S analysis, the Kruskal-Wallis test was used. Data were considered statistically significant when P values were ≤0.05.

### Data availability.

The 16S rRNA gene sequence data have been deposited in the European Bioinformatics Institute (EBI) database under accession no. PRJEB32443, along with the metatranscriptomic data under ArrayExpress accession number E-MTAB-7943.
