# Supplementary material for: Social-Stress-Responsive Microbiota Induces Stimulation of Self-Reactive Effector T Helper Cells
Source: mSystems. 2019 May 14;4(4):e00292-18. doi: 10.1128/mSystems.00292-18 (PMC6517692; doi:10.1128/mSystems.00292-18)
Supplement: FIG S1 [file mSystems.00292-18-sf001.pdf]

Figure 1S

A

Chronic social defeat (SD) model

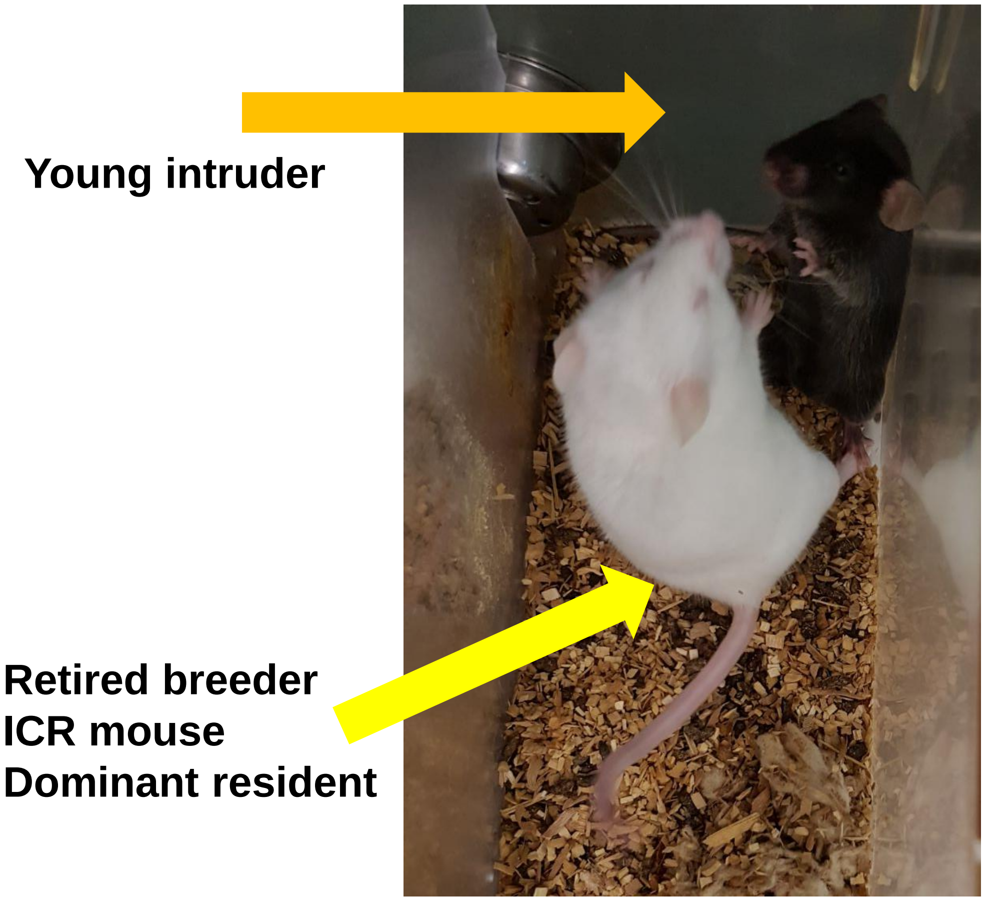

B

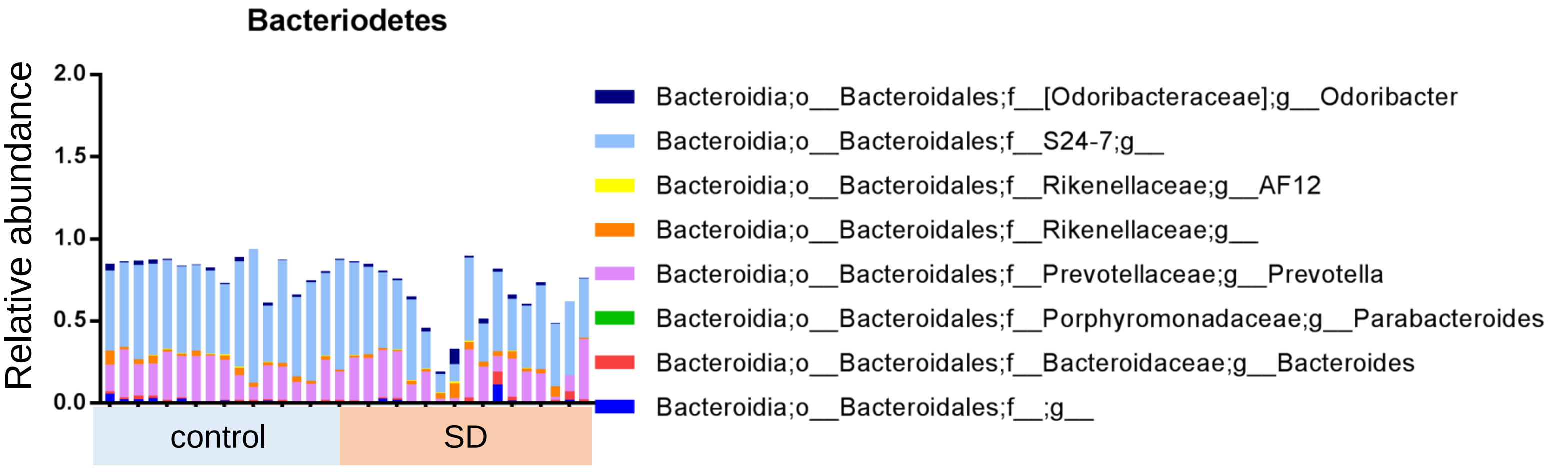
Bacteriodetes
Relative abundance
2.0
1.5
1.0
0.5
0.0
control
SD
Bacteroidia;o__Bacteroidales;f__[Odoribacteraceae];g__Odoribacter
Bacteroidia;o__Bacteroidales;f__S24-7;g__
Bacteroidia;o__Bacteroidales;f__Rikenellaceae;g__AF12
Bacteroidia;o__Bacteroidales;f__Rikenellaceae;g__
Bacteroidia;o__Bacteroidales;f__Prevotellaceae;g__Prevotella
Bacteroidia;o__Bacteroidales;f__Porphyromonadaceae;g__Parabacteroides
Bacteroidia;o__Bacteroidales;f__Bacteroidaceae;g__Bacteroides
Bacteroidia;o__Bacteroidales;f__;g__

C

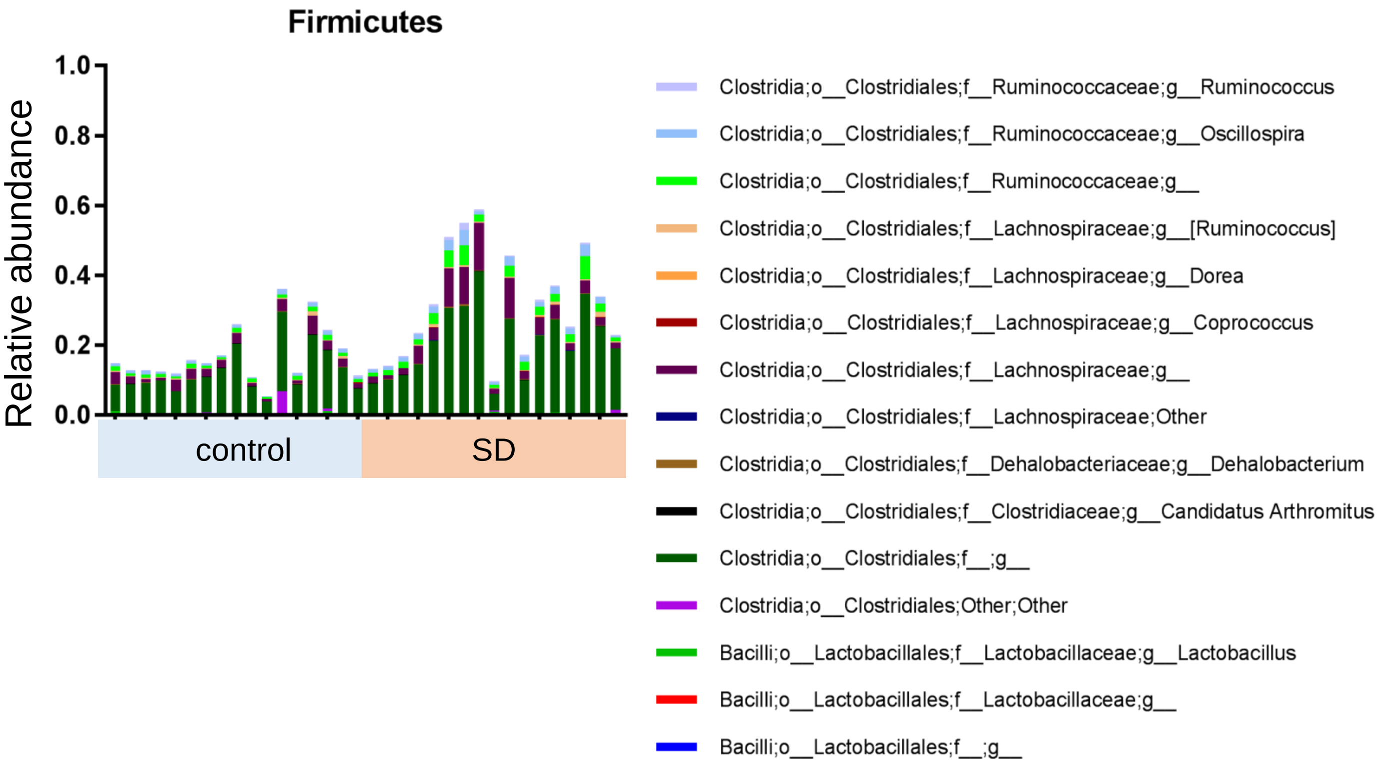
Firmicutes
Relative abundance
1.0
0.8
0.6
0.4
0.2
0.0
control
SD
Clostridia;o__Clostridiales;f__Ruminococcaceae;g__Ruminococcus
Clostridia;o__Clostridiales;f__Ruminococcaceae;g__Oscillospira
Clostridia;o__Clostridiales;f__Ruminococcaceae;g__
Clostridia;o__Clostridiales;f__Lachnospiraceae;g__[Ruminococcus]
Clostridia;o__Clostridiales;f__Lachnospiraceae;g__Dorea
Clostridia;o__Clostridiales;f__Lachnospiraceae;g__Coprococcus
Clostridia;o__Clostridiales;f__Lachnospiraceae;g__
Clostridia;o__Clostridiales;f__Lachnospiraceae;Other
Clostridia;o__Clostridiales;f__Dehalobacteriaceae;g__Dehalobacterium
Clostridia;o__Clostridiales;f__Clostridiaceae;g__Candidatus Arthromitus
Clostridia;o__Clostridiales;f__;g__
Clostridia;o__Clostridiales;Other;Other
Bacilli;o__Lactobacillales;f__Lactobacillaceae;g__Lactobacillus
Bacilli;o__Lactobacillales;f__Lactobacillaceae;g__
Bacilli;o__Lactobacillales;f__;g__

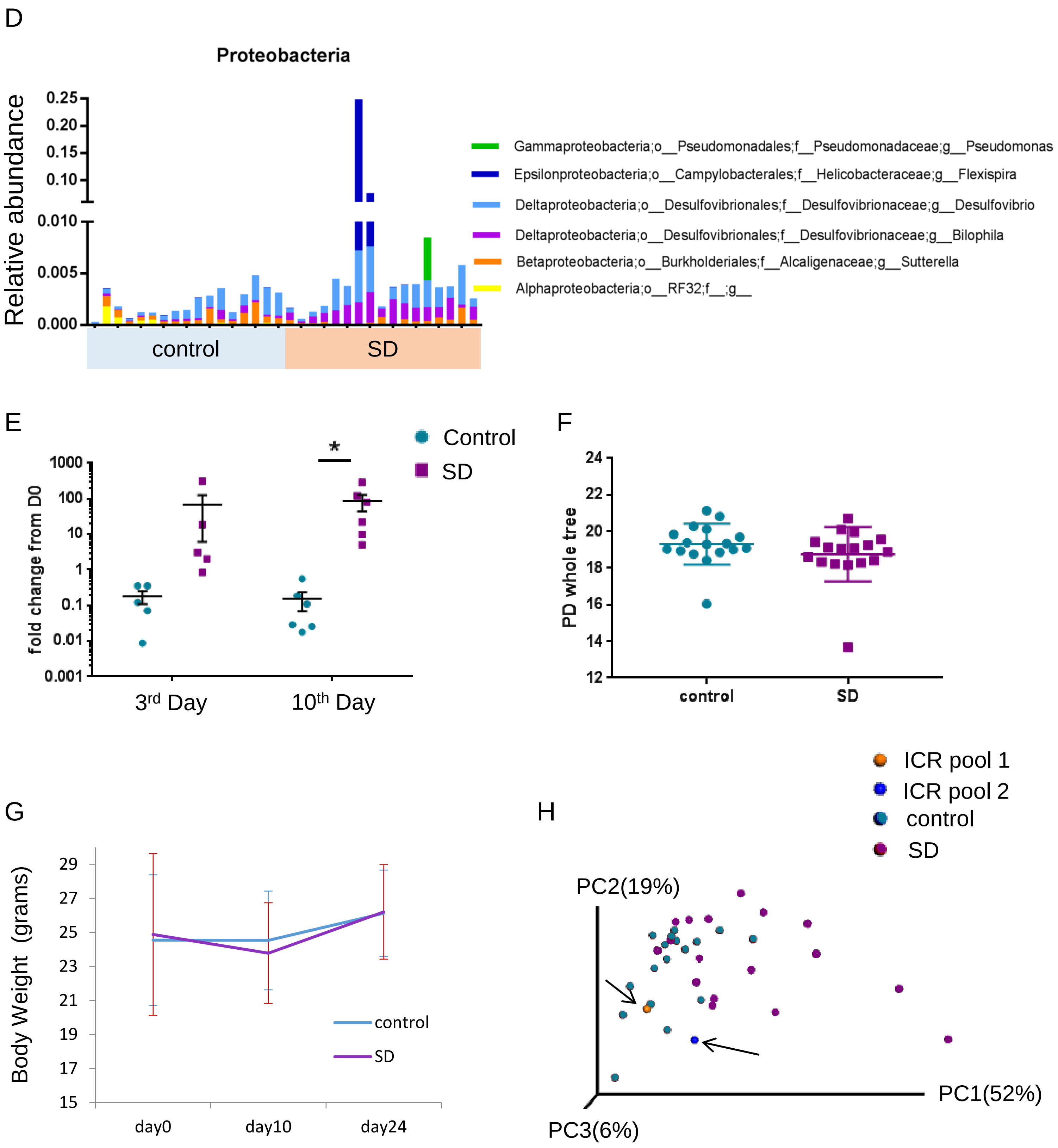

D
Proteobacteria
Relative abundance
0.25
0.20
0.15
0.10
0.010
0.005
0.000
control
SD
Gammaproteobacteria;o__Pseudomonadales;f__Pseudomonadaceae;g__Pseudomonas
Epsilonproteobacteria;o__Campylobacterales;f__Helicobacteraceae;g__Flexispira
Deltaproteobacteria;o__Desulfovibrionales;f__Desulfovibrionaceae;g__Desulfovibrio
Deltaproteobacteria;o__Desulfovibrionales;f__Desulfovibrionaceae;g__Bilophila
Betaproteobacteria;o__Burkholderiales;f__Alcaligenaceae;g__Sutterella
Alphaproteobacteria;o__RF32;f__;g__
E
Control
SD
fold change from D0
1000
100
10
1
0.1
0.01
0.001
*
3rd Day
10th Day
F
PD whole tree
24
22
20
18
16
14
12
control
SD
G
Body Weight (grams)
29
27
25
23
21
19
17
15
day0
day10
day24
control
SD
H
ICR pool 1
ICR pool 2
control
SD
PC2(19%)
PC1(52%)
PC3(6%)

I

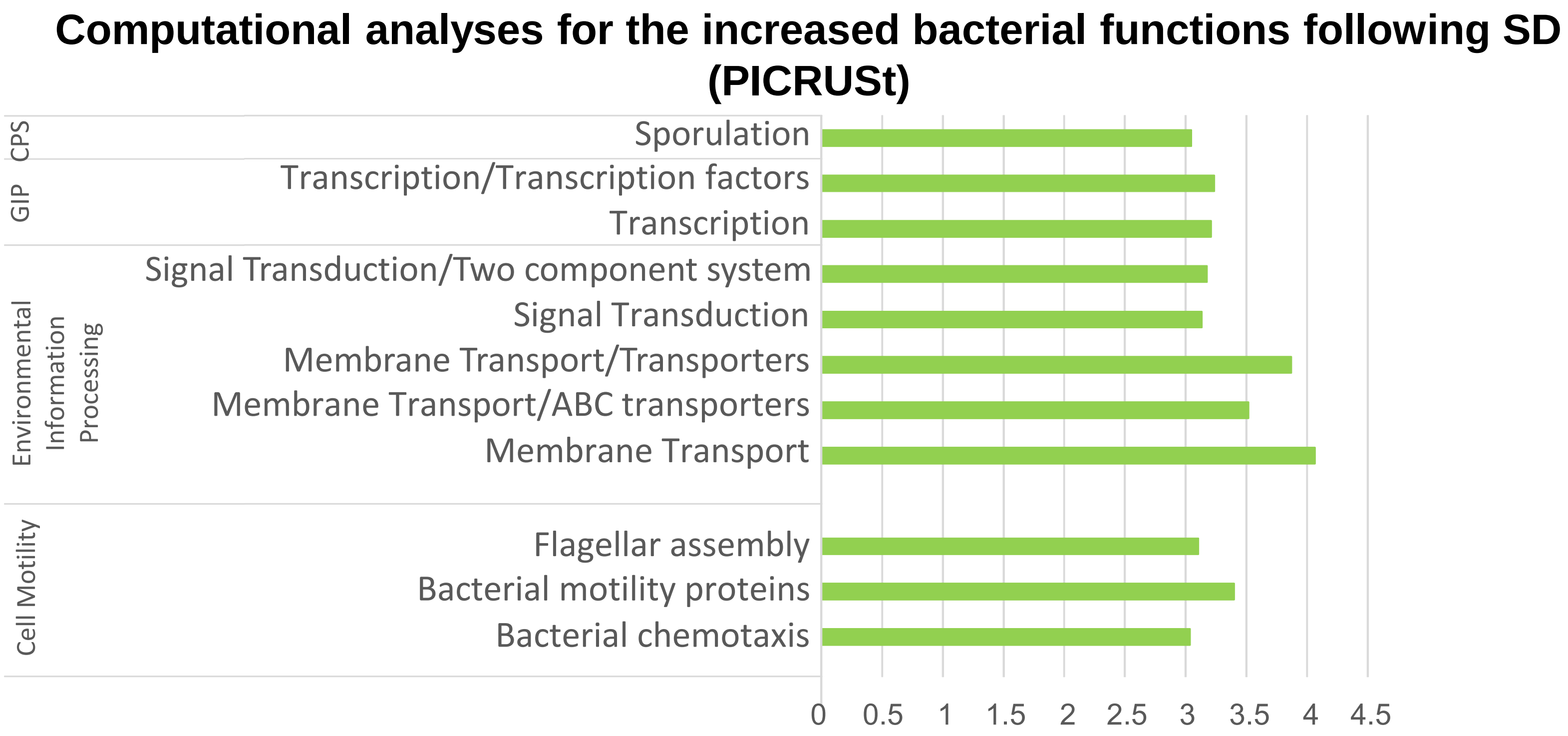

J

YALI0B09867p
fhu operon transcription regulator
Transposase putative
Negative regulator of genetic competence sporulation and motility
PF14266 domain protein
9-O-acetyl_N_acetylneuraminate esterase
UPF0297 protein BACPEC_00725
flagellar protein FliS, partial
flagellar biosynthesis protein FliS
chromosomal replication initiator protein DnaA
flagellar hook-associated protein FlgK
Activator of (R)-2-hydroxyglutaryl-CoA dehydratase
DTDP-glucose 4,6-dehydratase (Fragment)
DAK2 domain fusion protein YloV
Cysteine synthase
