# Supplementary material for: Social-Stress-Responsive Microbiota Induces Stimulation of Self-Reactive Effector T Helper Cells
Source: mSystems. 2019 May 14;4(4):e00292-18. doi: 10.1128/mSystems.00292-18 (PMC6517692; doi:10.1128/mSystems.00292-18)
Supplement: TABLE S1 [file mSystems.00292-18-st001.pdf]

Supplementary Tables:

Table S1

INCREASE					
# of Exp	OTU	P	Control mean	SD mean	Fold change
3 Exp	p__Proteobacteria|c__Deltaproteobacteria |o__Desulfovibrionales|f__Desulfovibrionaceae|g__Bilophila|s	3.0E-05	0.021%	0.134%	6.44
	p__Firmicutes|c__Clostridia|o__Clostridiales| f__Ruminococcaceae|g__|s__	3.3E-05	0.925%	2.592%	2.80
	p__Firmicutes|c__Clostridia|o__Clostridiales| f__Ruminococcaceae|g__Ruminococcus|s__	4.1E-04	0.214%	0.526%	2.46
	p__Firmicutes|c__Clostridia|o__Clostridiales| f__Ruminococcaceae|g__Oscillospira|s__	4.1E-04	0.765%	1.771%	2.32
	p__Deferribacteres|c__Deferribacteres|o__Deferribacterales |f__Deferribacteraceae|g__Mucispirillum|s__schaedleri	0.001	0.264%	0.920%	3.48
	p__Firmicutes|c__Clostridia|o__ Clostridiales|f__|g__|s__	0.003	11.57%	20.85%	1.80
	p__Firmicutes|c__Clostridia|o__Clostridiales| f__Dehalobacteriaceae|g__Dehalobacterium|s__	0.023	0.060%	0.142%	2.35
DECREASE					
# of Exp	OTU	P	Control mean	SD mean	Fold change
3 Exp	p__Bacteroidetes|c__Bacteroidia|o__Bacteroidales| f__S24-7|g__|s__	4.7E-04	54.910 %	38.329%	1.43
	p__Firmicutes|c__Bacilli|o__Lactobacillales| f__Lactobacillaceae|g__Lactobacillus|s__	0.004	0.363%	0.143%	2.53
	p__Proteobacteria|c__Alphaproteobacteria|o__RF32| f__|g__|s__	0.031	0.025%	0.003%	8.46
