# Supplementary material for: Social-Stress-Responsive Microbiota Induces Stimulation of Self-Reactive Effector T Helper Cells
Source: mSystems. 2019 May 14;4(4):e00292-18. doi: 10.1128/mSystems.00292-18 (PMC6517692; doi:10.1128/mSystems.00292-18)
Supplement: TABLE S2 [file mSystems.00292-18-st002.pdf]

Table S2
A.

# of Exp	OTU	P	Control mean	SD mean	Fold change
	INCREASE				
Exp 1	p__Firmicutes|c__Bacilli|o__Bacillales| f__Staphylococcaceae|g__Jeotgalicoccus|s__psychrophilus	0.008	1.100%	2.550%	2.32
	p__Proteobacteria|c__Gammaproteobacteria| o__Xanthomonadales|f__Xanthomonadaceae|g__|s__	0.018	0.175%	0.715%	4.09
	p__Firmicutes|c__Bacilli|o__Lactobacillales| f__Enterococcaceae|g__Enterococcus|Other	0.028	0.196%	0.560%	2.86
	p__Firmicutes|c__Bacilli|o__Lactobacillales| f__Lactobacillaceae|g__|s__	0.037	0.000%	0.390%	N/A
	p__Firmicutes|c__Bacilli|o__Turicibacterales |f__Turicibacteraceae|g__Turicibacter|s__	0.040	0.021%	0.075%	3.60
	p__Proteobacteria|c__Alphaproteobacteria|o__Caulobacterales|f__Caulobacteraceae|g__Brevundimonas|s__diminuta	0.045	2.063%	5.650%	2.74
	p__Proteobacteria|c__Gammaproteobacteria |o__Enterobacteriales|f__Enterobacteriaceae|g__|s__	0.045	1.525%	3.505%	2.30
Exp 2	p__Firmicutes|c__Clostridia |o__Clostridiales|f__|g__|s__	0.004	15.258%	34.833%	2.28
	p__Firmicutes|c__Clostridia|o__Clostridiales |f__Lachnospiraceae|g__Coprococcus|s__	0.015	0.113%	0.242%	2.15
	p__Cyanobacteria|c__4C0d-2 |o__YS2|f__|g__|s__	0.021	0.008%	0.104%	12.50
	p__Firmicutes|c__Clostridia |o__Clostridiales|f__Lachnospiraceae|Other|Other	0.025	0.267%	0.704%	2.64
	p__Deferribacteres|c__Deferribacteres|o__Deferribacterales |f__Deferribacteraceae|g__Mucispirillum|s__schaedleri	0.037	1.971%	7.629%	3.87
	p__Firmicutes|c__Clostridia |o__Clostridiales|f__Ruminococcaceae|g Ruminococcus|s__	0.045	0.296%	0.596%	2.01
Exp 3	p__Firmicutes|c__Bacilli|o__Gemellales |f__Gemellaceae|g__|s__	0.022	0.000%	0.179%	N/A
	p__Proteobacteria|c__Epsilonproteobacteria |o__Campylobacterales|f__Helicobacteraceae|g__Helicobacter|	0.022	0.000%	0.488%	N/A
	p__Actinobacteria|c__Coriobacteriia |o__Coriobacteriales|f__Coriobacteriaceae|g__Adlercreutzia| s	0.026	0.013%	0.167%	13.33
	p__Cyanobacteria|c__4C0d-2|o__YS2|f__|g__|s__	0.033	0.013%	0.113%	9.00

# of Exp	OTU	P	Control mean	SD mean	Fold change
	DECREASE				
Exp 1	p__Bacteroidetes|c__Bacteroidia |o__Bacteroidales|f__Prevotellaceae|g__Prevotella|s__	0.010	3.563%	1.146%	3.11
	p__Bacteroidetes|c__Bacteroidia|o__Bacteroidales |f__Bacteroidaceae|g__Bacteroides|s__acidifaciens	0.016	0.483%	0.138%	3.52
	p__Proteobacteria|c__Alphaproteobacteria |o__RF32|f__|g__|s__	0.021	0.021%	0.000%	N/A
	p__Proteobacteria|c__Betaproteobacteria |o__Burkholderiales|f__Alcaligenaceae|g__Sutterella|s__	0.024	0.283%	0.079%	3.58
	p__Bacteroidetes|c__Bacteroidia |o__Bacteroidales|f__Rikenellaceae|g__AF12|s__	0.030	0.217%	0.050%	4.33
	p__Firmicutes|c__Erysipelotrichi|o__Erysipelotrichales |f__Erysipelotrichaceae|g__Allobaculum|s__	0.036	0.067%	0.013%	5.33
	p__Bacteroidetes|c__Bacteroidia |o__Bacteroidales|f__|g__|s__	0.037	1.263%	0.479%	2.63
	p__Firmicutes|c__Clostridia |o__Clostridiales|f__Peptococcaceae|g__|s__	0.045	0.054%	0.013%	4.33
	p__Bacteroidetes|c__Bacteroidia |o__Bacteroidales|Other|Other|Other	0.049	0.092%	0.029%	3.14

B.

INCREASE					
# of Exp	OTU	P	Control mean	SD mean	Fold change
Exp 1	p__Actinobacteria|c__Actinobacteria |o__Actinomycetales|f__Micrococcaceae|g__|s__	0.011	0.000%	0.650 %	N/A
	p__Proteobacteria|c__Alphaproteobacteria |o__Caulobacterales|f__Caulobacteraceae |g__Brevundimonas|s__diminuta	0.022	0.088%	0.320 %	3.66
Exp 2	p__Bacteroidetes|c__Bacteroidia|o__Bacteroidales |f__Bacteroidaceae|g__Bacteroides|s__	0.006	0.104%	1.358 %	13.04
	p__Firmicutes|c__Clostridia|o__Clostridiales |f__Dehalobacteriaceae|g__Dehalobacterium|s__	0.006	0.154%	0.663 %	4.30
	p__Proteobacteria|c__Epsilonproteobacteria |o__Campylobacterales|f__Helicobacteraceae |g__Helicobacter|	0.007	0.000%	0.725 %	N/A
	p__Bacteroidetes|c__Bacteroidia|o__Bacteroidales |f__Bacteroidaceae|g__Bacteroides|s__acidifaciens	0.008	0.046%	0.492 %	10.73
	Unassigned|Other|Other|Other|Other|Other|Other	0.008	0.146%	2.317 %	15.89
	p__Actinobacteria|c__Coriobacteriia|o__Coriobacteriales |f__Coriobacteriaceae|g__Adlercreutzia|s__	0.009	0.025%	0.125 %	5.00
	p__Firmicutes|c__Clostridia|o__Clostridiales |f__Clostridiaceae|g__Candidatus Arthromitus|s__	0.036	0.096%	0.354 %	3.70
DECREASE					
# of Exp	OTU	P	Control mean	SD mean	Fold change
Exp 1	p__Deferribacteres|c__Deferribacteres|o__Deferribacterales |f__Deferribacteraceae|g__Mucispirillum|s__schaedleri	0.028	1.113%	0.255 %	4.36
