# Supplementary figures and images for: Social-Stress-Responsive Microbiota Induces Stimulation of Self-Reactive Effector T Helper Cells
Source: mSystems. 2019 May 14;4(4):e00292-18. doi: 10.1128/mSystems.00292-18 (PMC6517692; doi:10.1128/mSystems.00292-18)

Figure 2S

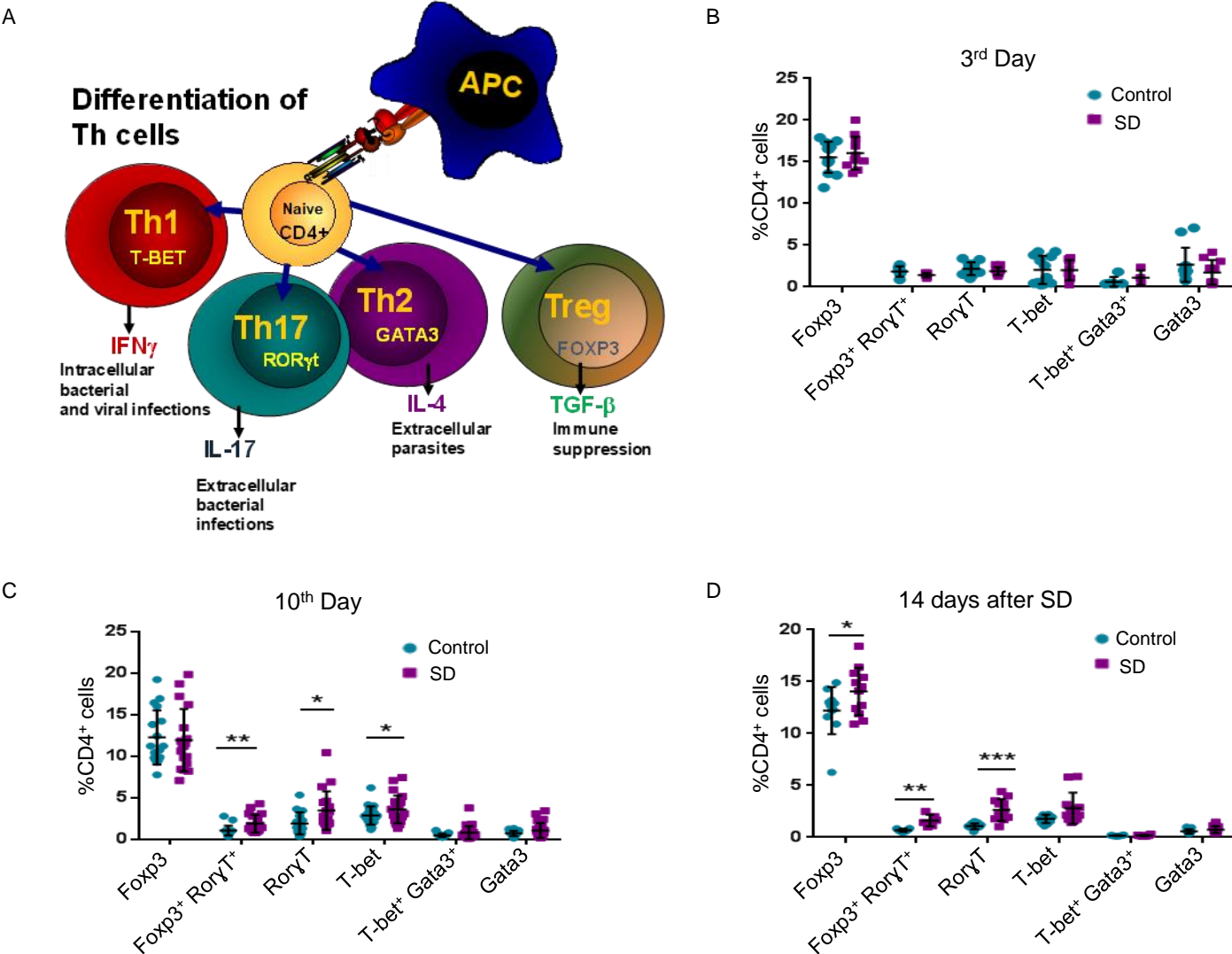

E

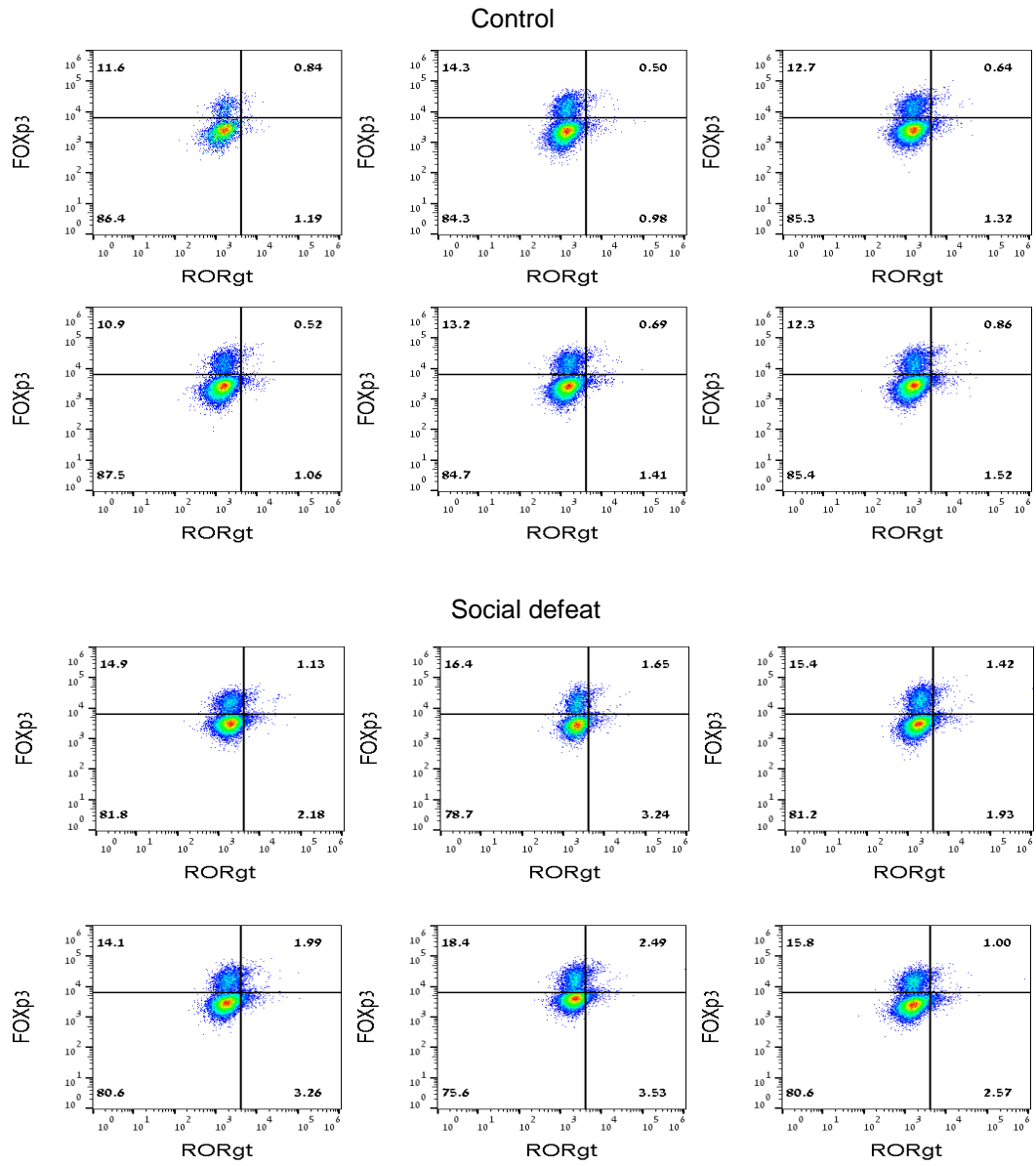

Supplement: FIG S2 [file mSystems.00292-18-sf002.pdf]

Figure S3

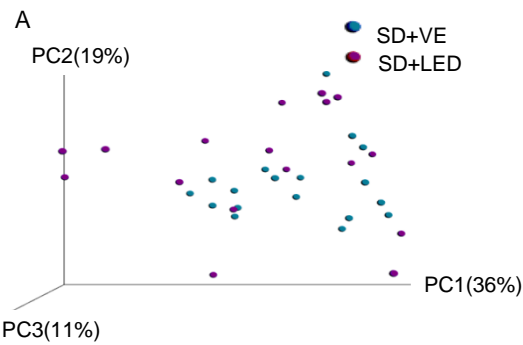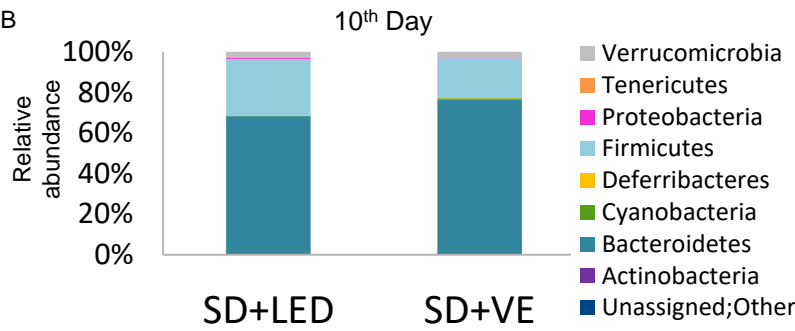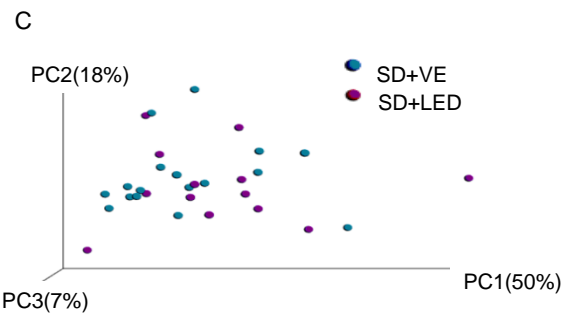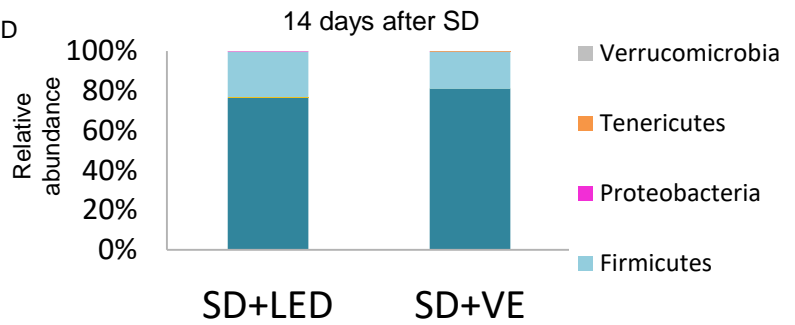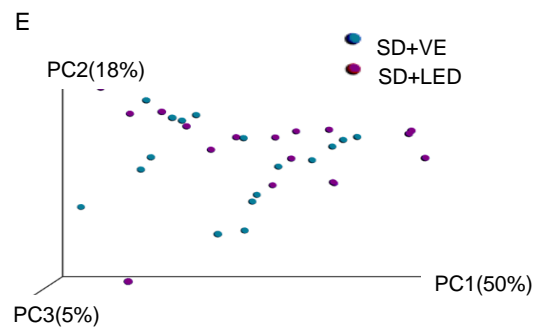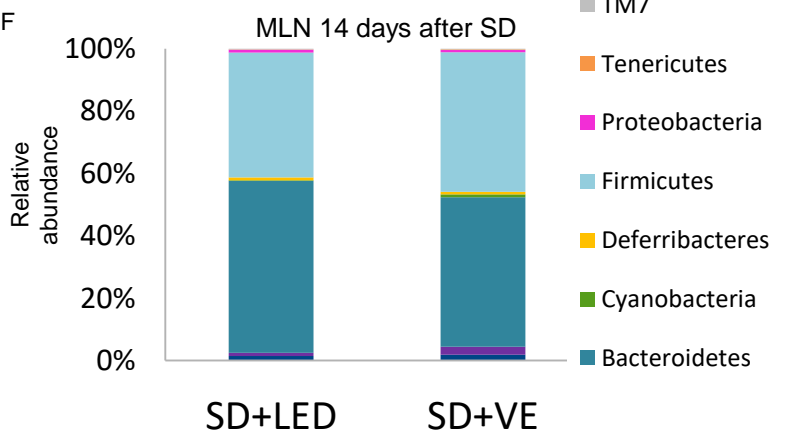

Supplement: FIG S3 [file mSystems.00292-18-sf003.pdf]
