# Supplementary material for: Social-Stress-Responsive Microbiota Induces Stimulation of Self-Reactive Effector T Helper Cells
Source: mSystems. 2019 May 14;4(4):e00292-18. doi: 10.1128/mSystems.00292-18 (PMC6517692; doi:10.1128/mSystems.00292-18)
Supplement: TABLE S3 [file mSystems.00292-18-st003.pdf]

Table S3

<table>
<tr><th colspan="6">INCREASE</th></tr>
<tr><th># of Exp</th><th>OTU</th><th>P</th><th>Control mean</th><th>SD mean</th><th>Fold change</th></tr>
<tr><td rowspan="4">Exp 1</td><td>p__Proteobacteria|c__Gammaproteobacteria |o__Enterobacteriales|f__Enterobacteriaceae|g__Proteus|s__</td><td>0.002</td><td>0.000%</td><td>8.538%</td><td>N/A</td></tr>
<tr><td>p__Firmicutes|c__Bacilli|o__Lactobacillales |f__Streptococcaceae|g__Lactococcus|s__garvieae</td><td>0.007</td><td>0.000%</td><td>0.213%</td><td>N/A</td></tr>
<tr><td>p__Firmicutes|c__Bacilli|o__Lactobacillales |f__Lactobacillaceae|g__Lactobacillus|s__</td><td>0.016</td><td>2.321%</td><td>38.033%</td><td>16.39</td></tr>
<tr><td>p__Cyanobacteria|c__4C0d-2|o__YS2|f__|g__|s__</td><td>0.056</td><td>0.000%</td><td>0.025%</td><td>N/A</td></tr>
<tr><th colspan="6">DECREASE</th></tr>
<tr><th># of Exp</th><th>OTU</th><th>P</th><th>Control mean</th><th>SD mean</th><th>Fold change</th></tr>
<tr><td rowspan="3">Exp 1</td><td>p__Firmicutes|c__Clostridia|o__Clostridiales |f__Ruminococcaceae|g__Ruminococcus|s__</td><td>0.004</td><td>0.888%</td><td>0.117%</td><td>7.61</td></tr>
<tr><td>p__Bacteroidetes|c__Bacteroidia |o__Bacteroidales|f__S24-7|g__|s__</td><td>0.010</td><td>51.667%</td><td>24.267%</td><td>2.13</td></tr>
<tr><td>p__Proteobacteria|c__Betaproteobacteria |o__Burkholderiales|f__Alcaligenaceae|g__Sutterella|s__</td><td>0.016</td><td>0.963%</td><td>0.313%</td><td>3.08</td></tr>
</table>
