# Supplementary material for: Social-Stress-Responsive Microbiota Induces Stimulation of Self-Reactive Effector T Helper Cells
Source: mSystems. 2019 May 14;4(4):e00292-18. doi: 10.1128/mSystems.00292-18 (PMC6517692; doi:10.1128/mSystems.00292-18)
Supplement: TABLE S4 [file mSystems.00292-18-st004.pdf]

Table S4

A.

# of Exp	OTU	P	SD+ve mean	SD+LED mean	Fold change
DECREASE (by LED209 administration)					
# of Exp	OTU	P	SD+ve mean	SD+LED mean	Fold change
3 Exp	p__Bacteroidetes|c__Bacteroidia |o__Bacteroidales|f__Rikenellaceae|g__AF12|s__	0.003	0.246%	0.117%	2.10
	p__Bacteroidetes|c__Bacteroidia |o__Bacteroidales|f__Prevotellaceae|g__Prevotella|s__	0.014	19.215%	15.638%	1.23
INCREASE					
# of Exp	OTU	P	SD+ve mean	SD+LED mean	Fold change
3 Exp	p__Tenericutes|c__Mollicutes|o__RF39|f__|g__|s__	0.016	0.024%	0.032%	1.35

B.

# of Exp	OTU	P	SD+Ve mean	SD+LED mean	Fold change
DECREASE (by LED209 administration)					
# of Exp	OTU	P	SD+Ve mean	SD+LED mean	Fold chang e
Exp1	p__Firmicutes|c__Bacilli|o__Bacillales |f__Planococcaceae|g__|s__	0.007	0.058%	0.000%	N/A
	p__Proteobacteria|c__Gammaproteobacteria |o__Pseudomonadales |f__Moraxellaceae|g__Acinetobacter|Other	0.022	0.075%	0.000%	N/A
Exp2	p__Firmicutes|c__Clostridia|o__Clostridiales |f__Ruminococcaceae|Other|Other	0.013	0.413%	0.096%	4.31
	Unassigned|Other|Other|Other|Other|Other|Other	0.016	1.246%	0.464%	2.68
Exp3	p__Bacteroidetes|c__Bacteroidia |o__Bacteroidales|f__Rikenellaceae|g__AF12|s__	0.009	0.184%	0.030%	6.22
	p__Actinobacteria|c__Actinobacteria |o__Actinomycetalesf__Propionibacteriaceae |g__Propionibacterium|s__acnes	0.013	0.259%	0.016%	15.80
	p__Bacteroidetes|c__Bacteroidia |o__Bacteroidales|f__|g__|s__	0.016	0.662%	0.197%	3.37
	p__Firmicutes|c__Bacilli|o__Lactobacillales |f__Lactobacillaceae|g__Lactobacillus|s__	0.047	2.292%	1.413%	1.62
INCREASE					
# of Exp	OTU	P	SD+Ve mean	SD+LED mean	Fold chang e
Exp1	p__Bacteroidetes|c__Bacteroidia|o__Bacteroidal es |f__Bacteroidaceae|g__Bacteroides|s__acidifacie ns	0.046	0.067%	0.304%	4.56
Exp2	p__Proteobacteria|c__Betaproteobacteria |o__Burkholderiales|f__Alcaligenaceae|g__Sutte rella|s__	0.046	0.019%	0.139%	7.29
Exp3	p__Proteobacteria|c__Betaproteobacteria |o__Burkholderiales|f__Alcaligenaceae|g__Sutte rella|s__	0.016	0.059%	0.233%	3.94
